# Valproic acid (VPA) in patients with refractory advanced cancer: a dose escalating phase I clinical trial

**DOI:** 10.1038/sj.bjc.6603851

**Published:** 2007-06-19

**Authors:** A Atmaca, S-E Al-Batran, A Maurer, A Neumann, T Heinzel, B Hentsch, S E Schwarz, S Hövelmann, M Göttlicher, A Knuth, E Jäger

**Affiliations:** 1 II. Medizinische Klinik/Onkologie, Krankenhaus Nordwest, Steinbacher Hohl 2-26, D-60488 Frankfurt am Main, Germany; 2 Topotarget Germany AG, 60596 Frankfurt am Main, Germany; 3 Chemotherapeutisches Forschungsinstitut Georg-Speyer-Haus, 60596 Frankfurt am Main, Germany; 4 Institut für Biochemie und Biophysik, Universität Jena, 07743 Jena, Germany; 5 GSF-Forschungszentrum, Institut für Toxikologie, 85764 Neuherberg/München, Germany; 6 Department of Oncology, University Hospital Zürich, 8091 Zürich, Switzerland

**Keywords:** valproic acid, advanced cancer, dose-limiting toxicity, maximum tolerated dose, HDAC inhibitor

## Abstract

Altered histone deacetylase (HDAC) activity has been identified in several types of cancer. This study was designed to determine the safety and maximum tolerated dose (MTD) of valproic acid (VPA) as an HDAC inhibitor in cancer patients. Twenty-six pre-treated patients with progressing solid tumours were enrolled in dose-escalating three-patient cohorts, starting at a dose of VPA 30 mg kg^−1^ day^−1^. VPA was administered as an 1-h infusion daily for 5 consecutive days in a 21-day cycle. Neurocognitive impairment dominated the toxicity profile, with grade 3 or 4 neurological side effects occurring in 8 out of 26 patients. No grade 3 or 4 haematological toxicity was observed. The MTD of infusional VPA was 60 mg kg^−1^ day^−1^. Biomonitoring of peripheral blood lymphocytes demonstrated the induction of histone hyperacetylation in the majority of patients and downmodulation of HDAC2. Pharmacokinetic studies showed increased mean and maximum serum VPA concentrations >120 and >250 mg l^−1^, respectively, in the 90 and 120 mg kg^−1^ cohorts, correlating well with the incidence of dose-limiting toxicity (DLT). Neurotoxicity was the main DLT of infusional VPA, doses up to 60 mg kg^−1^ day^−1^ for 5 consecutive days are well tolerated and show detectable biological activity. Further investigations are warranted to evaluate the effectivity of VPA alone and in combination with other cytotoxic drugs.

Histone acetylation of nucleosomal histones plays an important role in gene expression, and consequently affects proper cell function, differentiation, and proliferation. The acetylation status of histones is determined by the activity of enzymes called histone acetyltransferase (HAT) and histone deacetylase (HDAC) (Davie, 1998; Kouzarides, 1999; Strahl and Allis, 2000). Altered HAT and HDAC activity has been identified in several types of cancer (Cress and Seto, 2000; Mahlknecht and Hoelzer, 2000; Timmermann *et al*, 2001), and a number of HDAC inhibitors have been characterised that inhibit tumour growth *in vitro* and *in vivo* (Marks *et al*, 2000a, 2000b; Takai *et al*, 2004).

Valproic acid (VPA, 2-propylpentanoic acid) is a well-established drug in the long-term therapy of epilepsy and has been safely used for over three decades. In addition, VPA acts as a specific inhibitor of class I HDACs and induces proteasomal degradation of HDAC2, leading to cellular differentiation, growth arrest, and death *in vivo* and *in vitro* (Göttlicher *et al*, 2001; Phiel *et al*, 2001; Krämer *et al*, 2003; Catalano *et al*, 2005).

Long-term treatment of patients with VPA for prevention of epileptic seizures is usually performed with doses of 15–30 mg kg^−1^ day^−1^ leading to serum levels of 0.3–0.9 mM. With these doses, the most common adverse effects were transient gastrointestinal symptoms, including anorexia, nausea, and vomiting in about 16% of the patients. Neurotoxic effects, for example, sedation, ataxia, and tremour occurred less frequently and usually improved upon dose reduction. Severe VPA-related toxicity has been reported to involve the liver, pancreas, and haematopoietic system (Loscher, 1999).

This study was conducted to determine the maximum tolerated dose (MTD) and toxicity of infusional VPA applicated in two doses daily in a 5-day schedule. A secondary aim of the study was the evaluation of the clinical and biological response to VPA treatment, by monitoring the acetylation status of histones and the HDAC inhibition in the treated individuals.

## PATIENTS AND METHODS

### Patient eligibility

Patients with histologically confirmed progressive, advanced stage malignant disease without any options of standard treatment, who received at least one prior palliative chemotherapy, were considered eligible for the study. Further study criteria were Karnofsky performance status >60, age >18 years, life expectancy >12 weeks, measurable or evaluable disease, no major surgery within 4 weeks before study entry, and no concurrent anti-cancer treatment within the last 30 days.

Concurrent uncontrolled medical illness like congestive heart failure or unstable angina pectoris, previous history of myocardial infarction within 1 year from study entry, uncontrolled hypertension or high-risk uncontrolled arrhythmias, history of significant neurological, psychiatric, or addictive disorders, active peptic ulcer, unstable diabetes mellitus, active uncontrolled infection, bleeding disorders, hepatic or pancreatic disease, or severe renal function impairment were exclusion criteria.

Patients were required to have the following laboratory values, obtained within 14 days of study participation: granulocytes >2500 *μ*l^−1^, platelets >100 000 *μ*l^−1^, haemoglobin >8g dl^−1^, ASAT and ALAT <2.5 UNL, a-amylase <130 U l^−1^, lipase <300 U l^−1^, and creatinine <2 mg dl^−1^.

Pregnant or lactating women were excluded and patients of childbearing potential were required to have negative pregnancy test and were advised to take adequate precautions to prevent pregnancy. Participants gave written informed consent before they entered the study, which was approved by the Local Ethics Committee.

### Trial design and treatment

Patients were enrolled to the study in cohorts of three patients for each dose level. Projected dose levels were 30, 60, 120, 180, 240, and 300 mg kg^−1^ per treatment day. The protocol was amended to include dose levels 75 and 90 mg kg^−1^ later.

Patients were planned to receive a total of 20 intravenous administrations of VPA on days 1–5 and days 22–26. The daily does of VPA was divided into two equal parts and each part was given as an intravenous infusion of 60-min duration (VPA was dissolved in isotonic NaCl 0.9 or 5% glucose solution at a concentration of 900 mg 100 ml^−1^). The first infusion was administered in the morning between 0800 and 1000, and the second in the evening between 2000 and 2200.

Dose escalation to the next dose level was possible, if all patients of the previous dose levels have reached the end of the first infusion cycle (treatment days 1–5) without dose-limiting toxicity (DLT). In case of DLT in one out of three patients, three additional patients were enrolled to the same dose level. If at least two out of six patients at the same dose level experienced DLT, this dose level was closed and the dose level of the previous cohort was defined as the MTD. No dose adjustments of the study medication were planned.

### Toxicity assessment

All toxicities were graded according to the National Cancer Institute (NCI) Common Toxicity Criteria (CTC) (version 2). The following toxicities were considered dose limiting: NCI-CTC grade 4 anaemia, neutropenia, thrombocytopenia, nausea, vomiting, or any other NCI-CTC grades 3 or 4 non-hematological toxicities.

Patients receiving at least one dose of VPA were considered evaluable for toxicity.

### Treatment evaluation

The pre-treatment evaluation included a complete history, a physical examination with a baseline KPS, laboratory studies (white blood cells and neutrophil count, platelets count, alkaline phosphatase, ASAT, ALAT, bilirubin, lipase, amylase, and serum creatinine), imaging studies for tumour measurement/evaluation, and ECG. During treatment physical examination, laboratory studies and toxicity assessment were done every day. Tumour measurement for evaluation of response and ECG were done on day 40, according to the RECIST criteria.

Patients were considered evaluable for biological and clinical response after receiving at least 5-day doses of VPA.

### Biomonitoring

Blood samples (10 ml heparinised peripheral blood and 5 ml serum) were taken before start of the first infusion, and 6 h later on days 1 and 22 for western blot analyses and flow cytometry. Blood samples were also obtained before the start of the first infusion on days 3, 5, 24, and 26.

Accumulation of hyperacetylated histones H3, H4, and HDAC2 expression were analysed in cell lysates of peripheral blood lymphoctes (PBL) by western blotting using antibodies directed against acetylated histones H3 and H4 (Upstate Biotechnology Inc., Lake Placid, NY, USA) and HDAC2 (sc7899, Santa Cruz Biotechnology Inc., Santa Cruz, CA, USA). Whole-cell lysates were prepared in denaturing SDS sample buffer and separated on 15% SDS–polyacrylamide gels.

Acetylation status of histone H4 was also analysed in PBL by flow cytometry with an antibody directed against H4 (T52).

### Pharmacokinetics

Blood samples for pharmacokinetic analyses of serum VPA concentration were taken before the start of first infusion on every treatment day. Additional samples were obtained on days 4 and 25 immediately after the end of the first infusion (in the morning) and in some cases, before start of the second infusion (in the evening).

## RESULTS

### Patients

Twenty-six patients were enrolled in the study. All patients were assessable for safety and toxicity analysis. Eighteen patients were assessable for clinical response. One patient (in the 60 mg kg^−1^ dose level) discontinued treatment early for reasons other than toxicity (early tumour progression) and was replaced. The majority of patients were heavily pre-treated. The patient characteristics are outlined in Table 1.

### Dose escalation

VPA dose levels were escalated from 30 mg kg^−1^ over 60 to 120 mg kg^−1^, as planned (at the 30 mg kg^−1^ dose level, an additional cohort was opened as one patient had DLT, but retrospective analysis showed that this adverse event was most probably related to cerebral metastases and not to VPA). After >2 out of six patients at the 120 mg kg^−1^ dose level had DLT, the protocol was amended to include an additional dose level of 90 mg kg^−1^. A further dose level, 75 mg kg^−1^, was included per amendment, as there was DLT in >2 out of six patients at the 90 mg kg^−1^ dose level. After two out of five patients had DLT at the 75 mg kg^−1^ dose levels, 60 mg kg^−1^ was defined as MTD. The dose escalation schedule is outlined in Table 2.

Fifteen patients received all planned infusions of VPA, and in nine patients, the treatment was stopped early due to toxicity and in two patients due to rapid tumour progression. One patient refused further treatment after the first infusion cycle due to personal reasons.

### Toxicity

The most common type of toxicity in our study was neurological, occurring in almost all patients in a dose-dependent manner. Eight patients experienced a DLT. Neurocognitive impairment in form of confusion or disorientation represented the DLT in seven of these eight patients.

Five patients had neurovisual or neuroacustical side effects, but of lower grade. Also grade 1 or 2 vertigo was observed in five out of 26 patients. Somnolence occurred in 21 out of 26 patients and was dose limiting in two patients (both in the 120 mg cohort). All neurological side effects, whether dose limiting or not, resolved completely after discontinuation of the treatment. The toxicities are outlined in Table 3.

Haematological and metabolic toxicities were rare and mild (leucopoenia and thrombocytopenia). Gastrointestinal toxicity was also of lower grade, with 13 patients suffering from grade 1 or 2 nausea and/or vomiting. No relevant general toxicity was observed, except that five patients had fatigue, and in two patients, it was of grade 3 (dose-limiting).

### Clinical response

Eighteen (69%) of 26 patients were evaluable for response. No objective responses were observed. Two patients (one patient with non-small-cell lung cancer and one additional patient with colorectal cancer) had stable disease lasting 3 and 5 months, respectively. Both of the patients were previously treated and had rapid disease progression under the prior cytotoxic treatment.

### Pharmacokinetic studies

**Figure 1:**
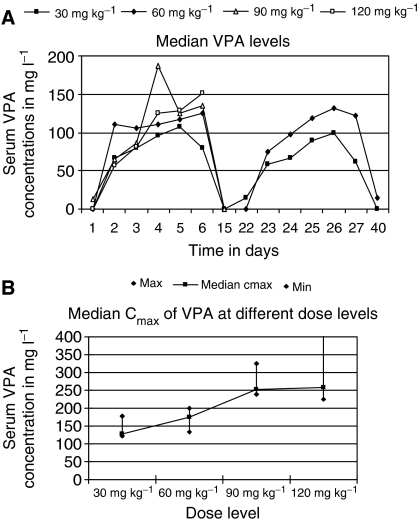
(**A**) Median VPA concentrations in different dose level cohorts (30 mg kg^−1^ (*n*=6 patients), 60 mg kg^−1^ (*n*=4 patients), 90 mg kg^−1^ (*n*=6 patients), and 120 mg kg^−1^ (*n*=4 patients)) and (**B**) median maximum serum VPA concentrations (cmax) at different dose levels.

At the 30 and 60 mg kg^−1^ dose levels, the median baseline VPA concentrations were in the range, normally achieved during anti-epileptic therapy (50–120 mg l^−1^; Figure 1A). No DLT was observed among the patients treated in these dose levels. In contrast, median VPA concentrations regularly exceeded 120 mg l^−1^ at the 90 and 120 mg kg^−1^ dose levels, with a tendency to higher concentration towards the end of the 5-day treatment correlating with the incidence of DLTs.

Figure 1B shows the median maximum concentrations of VPA at different dose levels. At the 30 and 60 mg kg^−1^ dose levels, the maximum serum concentration of VPA did not exceed 200 mg l^−1^, and at the 90 and 120 mg kg^−1^ dose levels, median maximum serum concentrations of VPA were above 200 mg l^−1^ with individual concentrations up to 500 mg l^−1^, indicating that also the high maximum VPA concentrations may contribute to the occurrence of DLTs.

### Biomonitoring

**Figure 2:**
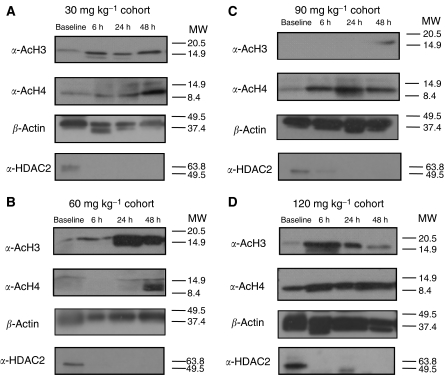
Western blot analyses of peripheral blood lymphocytes from patients treated with VPA at different dose levels (**A**) 30, (**B**) 60, (**C**) 90, and (**D**) 120 mg kg^−1^ with antibodies directed against acetylated histones H3, H4, and HDAC-2. As a loading control for the histone protein, parallel gels were run and stained for *β*-actin. Analysis was performed at baseline after 6, 24, and 48 h. In all dose levels, an increase of histone hyperacetlyation and downregulation of HDAC-2 were observed under VPA treatment. Molecular weights (MW) markers are given in kDa.

An increase of histone hyperacetylation was observed in 12 (75%) out of 16 tested patients under VPA treatment, with either H3-hyperacetylation and/or H4-hyperacetylation of PBL, analysed by Western blot. Figure 2 shows representative results of western blot analyses in four patients treated at different dose levels. Histone hyperacetylation did not seem to be dose dependent. Four patients had no detectable biological activity, two of whom were treated at 120 mg kg^−1^, one at 30, and one at 60 mg kg^−1^ dose level.

Monitoring the histone acetylation of PBMC with T52 via FACS also showed an increase of the acetylation status of PBL during VPA treatment in 10 out of 10 patients tested.

The expression of HDAC2 was analysed by western blot in four patients (at dose levels 30, 60, 90, and 120 mg kg^−1^). Downregulation of HDAC2 was observed in all patients tested (Figure 2).

## DISCUSSION

HDAC inhibition represents an interesting mechanism of anti-cancer treatment. Several HDAC inhibitors, including phenylbutyrate, depsipeptide, and suberoylanilide hydroxamic acid (SAHA) are currently under investigation in clinical studies (Carducci *et al*, 2001; Sandor *et al*, 2002; Kelly *et al*, 2003, 2005). The compounds apparently differ in their activity and safety profiles from each other and from VPA. The fact that VPA has been safely used in the long-term therapy of patients with epilepsy over decades clearly represents an advantage of VPA.

Our study was conducted to determine the MTD and define the DLT of intravenous VPA given as an 1-hour infusion twice daily for 5 consecutive days in a 21-day cycle in patients with advanced solid tumours. Furthermore, we conducted a pharmacokinetic analysis to assess the correlation between the side effects observed and the VPA serum concentration reached at each dose level. The study, in addition, aimed to investigate whether intravenous VPA induces hyperacetylation *in vivo*, as assessed in patients’ PBL. To our knowledge, this study represents the first report of a clinical trial with VPA as a HDAC inhibitor in patients with solid tumours.

The treatment was well tolerated in patients treated with VPA 30 or 60 mg kg^−1^ day^−1^. These patients had median VPA concentrations ranging between 50 and 120 mg l^−1^, which were slightly higher than those observed in patients receiving VPA during anti-epileptic therapy (Loscher, 1999). On the other hand, severe neurological side effects including disorientation, confusion, and somnolence dominated the toxicity profile and were dose limiting in most of the patients who received VPA at 90 or 120 mg kg^−1^ day^−1^. In these patients, median and maximum VPA serum concentrations were >120 and 200 mg l^−1^, respectively. After an additional dose level at 75 mg kg^−1^ day^−1^ was also found toxic (DLT in two out of five patients), the MTD of infusional VPA was defined as 60 mg kg^−1^ day^−1^ for 5 consecutive days. This dose is consistent with the results of a phase 1/2 study reported by Garcia-Manero *et al* (2006) in patients with advanced leukaemia receiving the combination of a fixed dose of 5-aza-2′-deoxycytidine and escalating doses of VPA for 10 days, where a daily dose of 50 mg kg^−1^ of VPA was found to be safe.

The availability of an oral formulation of VPA represents an additional advantage of VPA. We, however, preferred the intravenous over the oral use of VPA in the study because we expected that a proportion of the study patients may have gastrointestinal disturbances (due to the very advanced stages of disease) which may affect the uptake of VPA.

*In vitro* experiments by Yang and colleagues (Yang *et al*, 2005) demonstrated that high doses of VPA were needed to show hyperacetylation and antitumour effects in tumour cell lines. The amount of hyperacetylation was enhanced with increasing concentrations of VPA up to 10 mM (Yang *et al*, 2005). In consideration of these data, we chose an intermittent schedule of VPA in an attempt to be able to administer higher doses of VPA, as it would have been possible using the continuous dosing. The results, however, showed that we could not markedly increase the dose of VPA safely over that usually used in the long-term treatment.

The use of VPA in our study was associated with an increase of acetylated histones and a decrease of HDAC2 protein levels as assessed by western blot analysis and flow cytometry in peripheral blood lymphocytes of patients in all dose levels. This is important because it suggests that we could achieve detectable biological activity using the lower doses of VPA, which were found tolerable in the study. Notably, we did not evaluate the histone acetylation status of the tumours. In a recent study, however, the accumulation of acetylated histones in peripheral blood mononuclear cells after the administration of the HDAC inhibitor SAHA correlated well to the accumulation of acetylated histones in patients’ tumours as assessed by immunohistochemistry (Kelly *et al*, 2003).

Efficacy was a secondary end point of the trial. No radiological responses were observed. One patient with metastatic non-small-cell lung cancer and one additional patient with metastatic colorectal cancer had a stable disease, lasting 3 and 5 months, respectively. Both patients had documented rapid disease progression under their prior cytotoxic therapy, as was documented by subsequent CT scans. The stable disease achieved in these patients may, therefore, be related to VPA. Overall, the efficacy results of our study are, in fact, very difficult to interpret due to several aspects, including the small size and heterogeneity of the study population. Objective responses to HDAC inhibitors have been observed using SAHA in patients with lymphoma and bladder cancer (Kelly *et al*, 2003) or VPA in combination with all-trans retinoic acid in patients with acute leukaemia (Bug *et al*, 2005; Kuendgen *et al*, 2006). The activity of VPA in patients with solid tumours may be improved by patient selection based on biological parameters (e.g. HDAC2 overexpression) or by the combination of VPA with drugs that may act synergistic with HDAC inhibitors. Additive effects of VPA and mitomycin C could be demonstrated in adenocarcinoma cell lines and fresh tumour cells from patients with colon cancer *in vitro* by our group (Friedmann *et al*, 2006). Future studies are needed to investigate these issues.

## Figures and Tables

**Table 1:** Patient characteristics

	**No. of patients**	
**Patient characteristics**	**(*n*=26)**	**%**
*Age (years)*		
Median	62.5	
Range	39–75	
		
*Sex*		
Female	12	46
Male	14	64
		
*Karnofsky performance status*		
100	5	19
90	16	62
80	4	15
70	1	4
		
*Tumour type*		
Colorectal	10	38
Melanoma	4	15
Breast	2	8
Non-small-cell lung cancer	3	12
Prostate	1	4
Ovarian	1	4
Oesophageal	1	4
Ileum	1	4
Thymus	1	4
CUP	1	4
Myeloma	1	4
		
*No of prior palliative chemotherapies*		
1–2	10	38
3–4	12	46
>4	4	15
		
*Organs involved (metastatic)*		
1	7	27
2	10	38
⩾3	9	35

**Table 2:** Dose escalation schedule

**Cohort number**	**Dose (mg kg^−1^ body weight)**	**No. of patients**	**DLT**
1 and 2	30	6	0^a^
3	60	3	0
4 and 5	120	5	4^b^
6 and 7	90	6	4
8 and 9	75	5	2

DLT, dose-limiting toxicity.

a Symptoms were related to cerebral metastasis.

b One additional patient had a delayed adverse event which was later considered DLT during data monitoring, after the cohort was already extended.

**Table 3:** Common toxicity (*n*=26 patients)

	**Grade 1**	**Grade 2**	**Grade 3**	**Grade 4**
**Toxicity**	***n* (%)**	***n* (%)**	***n* (%)**	***n* (%)**
*Haematological*				
Leucopenia	3 (12)	1 (4)	—	—
Thrombocytopenia	2 (8)	—	—	—
Anaemia	—	—	—	—
				
*Gastrointestinal*				
Diarrhoea	2 (8)	—	—	—
Nausea/vomiting	9 (35)	4 (15)	—	—
Constipation	1 (4)	—	—	—
Anorexia	1 (4)	1 (4)	—	—
				
*Metabolic*				
Creatinine	—	—	—	—
AST	—	1 (4)	—	—
ALT	1 (4)	—	—	—
Lipase/amylase	1 (4)	—	—	—
				
*Neurological*				
Neurosensory	4 (15)	1 (4)	—	—
Neuromotor	—	—	—	—
Neurocortical (cognitive disturbance/confusion)	5 (19)	1 (4)	8 (31)	—
Vertigo	4 (25)	1 (4)	—	—
Neuroconstipation	—	—	1 (4)	—
Headache	2 (8)	—	—	—
Somnolence	15 (58)	4 (15)	2 (8)	—
				
*General*				
Fatigue	1 (4)	2 (8)	2 (8)	—
Pain	1 (4)	—	—	—
Fever	—	—	—	—
Skin	—	1 (4)	—	—
